# The Effect of a Comprehensive Corrective Exercise Program on Kyphosis Angle and Balance in Kyphotic Adolescents

**DOI:** 10.3390/healthcare10122478

**Published:** 2022-12-08

**Authors:** Gönül Elpeze, Günseli Usgu

**Affiliations:** 1 Kalyon Medical Center, Hasan Kalyoncu University, 27010 Gaziantep, Turkey; 2 Department of Physical Therapy and Rehabilitation, Faculty of Health Sciences, Hasan Kalyoncu University, 27010 Gaziantep, Turkey

**Keywords:** adolescent, kyphosis, posture, exercise, perception

## Abstract

This study aimed to investigate the effects of a comprehensive corrective exercise program on the kyphosis angle and balance in kyphotic adolescents. A total of 62 male adolescents (between the ages of 10 and 18, mean BMI 21.7 kg/m^2^) with a thoracic kyphosis (TK) angle of ≥ 50° were divided into three groups using the simple randomization method: CCEP (comprehensive corrective exercise program), TEP (thoracic exercise program) and control group. The CCEP program consisted of corrective exercises plus postural perception training (PPT). Exercise programs were applied for 40–50 min, 3 days a week for 12 weeks. The kyphosis angle was measured using a flexible ruler, and balance was assessed using the Romberg index obtained from pedobarography. After training, a highly significant reduction in the kyphosis angle was observed in the CCEP and TEP groups (*p* < 0.001). Comparison among the groups showed a greater reduction in the kyphosis angle in the CCEP group (*p* < 0.020). Postural perception improved in the CCEP group versus other groups (*p* < 0.001). Improvement of the Romberg index (balance) was found only in the CCEP group upon within-group comparison (*p* < 0.001), with no difference among the groups (*p* > 0.05). The use of postural perception in combination with corrective exercise programs for thoracic kyphosis represents a comprehensive approach, and PPT can increase the effectiveness of the intervention.

## 1. Introduction

Increased thoracic curvature of the spinal column is a postural disorder known as sagittal plane deformity. The normal thoracic kyphosis (TK) angle of the spine ranges from 20° to 40°, and TK angles of 45–50° are referred to as postural kyphosis, increased kyphosis or hyperkyphosis [1,2]. In children, reduced physical activity, rapid growth and poor lifestyle habits lead to weakness of the trunk muscles and dysfunctional deformities of the spine during a growth spurt [3]. Increased thoracic kyphosis is a deformity that can occur as a result of spinal misalignment in all age groups [4,5,6]. Hyperkyphosis affects 32% of adults and 60% of the elderly population [7]. Spinal deformities have been reported in 30% of school-age children [8]. In a study, screening of adolescents revealed improper posture in 58.85% and spinal deformities in 23.67% of children [9]. In children and adolescents, increased thoracic kyphosis (TK) may occur due to poor standing posture, sitting in front of a computer for long hours in an incorrect posture, and carrying heavy schoolbags [5,10,11]. Failure to correct spinal deformities in adolescence may cause static deformations in youth and adulthood [10,12]. Thus, early diagnosis and correct assessment in adolescence may impede different effects of postural and spinal deformities [12]. Additionally, with accelerated improvement of speed, coordination and movements, gains in muscular strength occur in adolescents aged 10 to 17 years [9,11], and during this stage, suitable treatment, rehabilitation and active exercise programs can be recommended [12]. 

The reported prevalence of poor posture in adolescents varies from 22 to 65% [8,13,14]. In adolescents, improper posture and poor postural awareness may become habitual over time [15]. Habitual incorrect posture has been shown to reduce the spine’s ability to perceive, maintain, and reposition neutral postures [16]. Poor postural awareness has been associated with habitual posture changes that place greater strain on supporting structures [16]. It has been reported that unawareness of non-ideal postures while standing and sitting can have serious long-term effects [17]. Studies have shown that persistent incorrect posture in adolescence may lead to severe postural disorders in adulthood [15]. Correspondingly, we think that it is important to correct improper posture and improve postural awareness in adolescence in order to avoid postural disorders in later life. There are a number of studies that applied training and exercise programs to correct postural disorders associated with increased TK [12,18,19]. However, these studies mostly focused on correcting poor posture rather than improving postural awareness. We believe that it would be more effective to provide postural awareness training first and then apply training and exercises to correct improper posture. To our knowledge, no such training has been used in former studies involving individuals with TK. Therefore, in this study, a training program (PPT) was applied to improve postural awareness in adolescents with TK.

A variety of methods are used for the treatment of kyphosis, including manual therapy, posture exercises, orthosis, taping, surgery and therapeutic exercises [20,21,22,23,24]. Exercises used for the treatment of TK aim to strengthen muscle groups and realign the spinal segments [12,19,25]. In some studies, stretching and strengthening exercises were applied only on the affected area in order to reduce TK [26]. Improvement in the kyphosis angle was reported in a study investigating the effectiveness of a manual therapy and an exercise program for the thoracic region [24]. Thoracic joint mobilization and extension exercises were reported to improve thoracic alignment [27]. In a study comparing the effects of manual therapy and mechanical massage in thoracic hyperkyphosis, both methods were found to be effective in improving TK angle [28]. The fact that any abnormality occurring in the thoracic spine may propagate in a chain reaction to involve other regions [2], along with the lack of controlled studies investigating the effectiveness of thoracic spine-specific approaches, has prompted researchers to examine the effectiveness of comprehensive exercise programs in thoracic kyphosis [6,12,29,30,31]. Seidi et al. reported less improvement in TK angle with local corrective exercise programs than with comprehensive corrective exercise programs [30]. Feng et al. reported a reduction in the kyphosis angle in adolescents with postural thoracic kyphosis participating in a corrective functional exercise program [6]. Randomized controlled studies have demonstrated that muscle strengthening exercises alone are less effective than corrective exercises in improving thoracic kyphosis [6,31]. Positive effects of a 12-week program including spinal mobility, strengthening and alignment on kyphosis were demonstrated in a study [12].

The increase in thoracic kyphosis may shift the body’s center of gravity away from the line of gravity. Accordingly, the balanced movements of the trunk may be impaired and greater muscle activation required to stabilize the spine [32]. In the literature, there are studies examining the effects of corrective, strengthening and flexibility exercise programs in improving balance in individuals with thoracic kyphosis, but most of these studies included older people because balance impairment is associated with an increased risk of falls [4,18,19,25,33,34,35,36,37,38]. There are few studies in the literature involving adolescents with thoracic kyphosis [6,39]. For this reason, this study was conducted with an adolescent population. Male adolescents were chosen for the current study because previous studies reported that boys aged 10–17 years have a significantly higher management rate of musculoskeletal problems than girls of the same age. The management rate of spine and trunk problems in boys shows a significant increase with each age group (aged 10–14 years and 15–17 years) [40,41]. Adolescent girls are more likely to drop out of exercise programs than boys [42]. Thus, we think that our study, since it includes male adolescents with thoracic kyphosis, can add valuable data to the literature. However, it has been reported that muscle strengthening exercises have limited effectiveness in correcting thoracic kyphosis when applied alone [6,30]. As such, we believe that it is important to apply strengthening exercises in combination with postural perception training for correcting TK in adolescents. However, there is no study in the literature that used a corrective exercise program together with postural perception training in kyphotic adolescents. Therefore, this study primarily aimed to investigate the effects of a CCEP on the kyphosis angle and balance in male adolescents with thoracic kyphosis. The second aim of the study was to compare the effects of a CCEP on the kyphosis angle and balance versus a TEP in male adolescents with TK.

## 2. Materials and Methods

### 2.1. Participants

This was a randomized, controlled study. The adolescents participating in the study were selected among the students of a private school (Erdem College) in Gaziantep, Turkey. Signed informed consent was obtained from the children and their parents for publication of their data. The study protocol was approved by the Ethics Committee for Non-Interventional Studies of Hasan Kalyoncu University Faculty of Health Sciences (2021/057).

**Figure 1:**
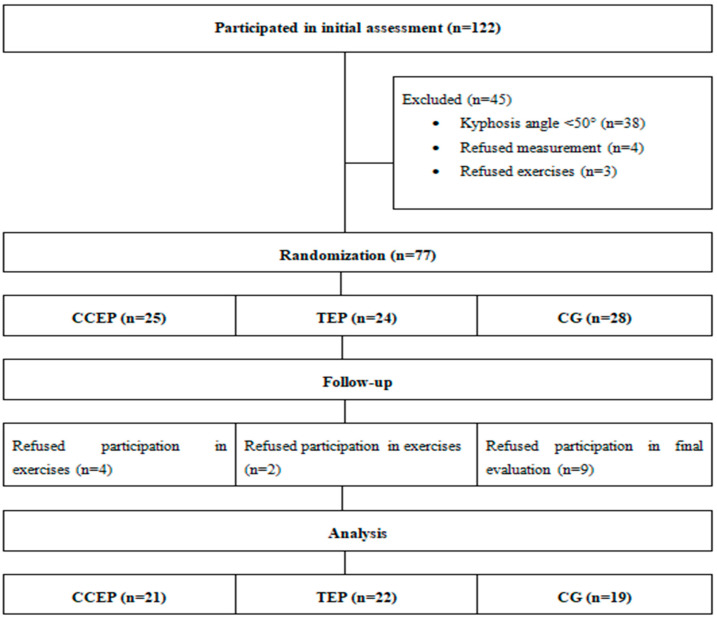
Study flow diagram.

Male adolescents aged 10 to 18 years with a kyphosis angle of ≥50° were included in the study. Individuals with rigid thoracic kyphosis, scoliosis with a Cobb angle of >10°, congenital spine deformities, disorders of the spine, pelvis, and shoulder, medical conditions hindering physical activity, and professional athletes were excluded. A total of 122 individuals who agreed to participate in the study were evaluated. Based on the results of initial assessment, 38 adolescents with a kyphosis angle of >50° were excluded. Seven other adolescents were excluded due to refusal to undergo further assessments (*n* = 4) and refusal to perform exercises (*n* = 3). The remaining 77 participants were randomly assigned to the CCEP group, TEP group or control group (CG) with no exercise intervention, using the simple randomization method. During the follow-up period, 4 adolescents from the CCEP group and 2 adolescents from the TEP group were excluded because they discontinued their exercise programs. Nine adolescents from the CG group were excluded due to failure to undergo final assessment. Ultimately, 62 subjects completed the study (Figure 1). The CG received the CCEP program at the end of 12 weeks. 

### 2.2. Outcome Measures

Measurements for thoracic kyphosis and balance were obtained before and after the study for all subjects. First, the thoracic kyphosis angle was measured using a smartphone inclinometer (Samsung, Clinometer Version 2.4, https://www.plaincode.com/products/clinometer/, Android 2.3.2+, 30 May 2016). The smartphone inclinometer was used to determine the kyphosis angle (≥50°) of the subjects, which was among the inclusion criteria of our study. We chose to use the smartphone inclinometer. Shahri et al. compared the Goniometer-Pro app to the Cobb angle method for the measurement of thoracic kyphosis. They found excellent correlation (r = 0.81, *p* = 0.000) for intra-rater (ICC = 0.88) and inter-rater reliability (ICC = 0.915). Both methods also showed good agreement [43]. Secondly, the thoracic kyphosis angle was measured using a flexible ruler. The data obtained with the flexible ruler was used to follow the changes in the kyphosis angle throughout the study with the authors’ joint agreement.

The flexible ruler (Flexicurve) is used to measure the curvatures of the thoracic and lumbar regions in the sagittal plane [44]. In a systemic review, Barrett et al. demonstrated that the strongest levels of evidence existed for reliability in support of the Debrunner kyphometer, Spinal Mouse and Flexicurve index, and, for validity supports, the arcometer and Flexicurve index [45]. Moderately strong correlations were reported between the Cobb angle and Flexicurve (r = 0.55–0.76) in many studies [46,47,48,49]. In this study, the subject was positioned in a neutral standing position for the TK measurement. Anatomical landmarks (T1-T12) were marked as described in the literature [44,46,49]. After the subject was asked to flex their neck, C7 was palpated, and the T1 spinous process underneath was marked. Self-adhesive skin markers were used to indicate spinous processes. The landmarks were marked by one physiotherapist to ensure standardization. The same physiotherapist obtained measurements from these locations with a smartphone inclinometer and flexible ruler to minimize human error while recording. Typically, palpation errors can occur in non-invasive measurements of the spine. However, there is evidence from studies that the experience of the assessor can minimize palpation errors [50,51,52]. We believe that the margin of error in our study was reduced due to the long years of experience of the physiotherapist (21 years) included as assessor in our study and due to the palpation and measurements being performed by a single assessor. For the protractor software of the smartphone inclinometer app to work, the short side of the phone was placed over the anatomical landmarks (T1-T3, T12) marked on the spine. The flexible ruler was molded to the shape of the curvature along the anatomical landmarks (T1-T12) using gentle pressure. Then, the ruler was transferred onto graph paper taking care not to distort its shape. Curvature contour was outlined with a pencil. The angular value of the curvature drawn on the paper was calculated using the following formula: arctan (B/X1) + arctan (B/X2), where B denotes the height of thoracic kyphosis and X1-X2 denotes the distance from T1-T12 points to the B point [44,46]. 

In pedobarography, data from static, dynamic and stabilometric measurements obtained on force platforms are used to evaluate balance [53]. The Romberg index (RI), one of the outputs of stabilometric measurement, is the ratio of the measurement value taken with eyes closed to that with eyes open. Higher RI values indicate balance impairment, and lower RI values imply improvement of balance. In our study, stabilometric measurements were obtained using the DIASU Digital Analysis System^®^ and Milletrix gait analysis software (DIASU, Sani Corporate via Giacomo Peroni 400 00131, Rome, Italy) to evaluate balance.

The PPT applied in the present study aims to improve postural awareness of the individual while sitting and standing and to increase the number of posture corrections by providing feedback on ideal spinal alignment. For the assessment of PPT, the participants were questioned on a weekly basis about the number of times they noticed and corrected their posture, and the average number/week was recorded. This procedure was repeated every week starting from the first week until the last week of the study. In this study, PPT was applied to the CCEP group only. 

### 2.3. Intervention

Individuals in the exercise groups received their respective exercise programs for 40–50 min, 3 days a week for 12 weeks. The duration and intensity of the exercise programs applied in our study were consistent with those reported in the literature [54] (see Appendix A). The number and total duration of exercises were identical among the groups. Exercises were applied for 3 days over a period 12 weeks under the supervision of a physiotherapist. Interventions consisted of initial 10-min warm up exercises for large muscle groups, continued with program-specific exercises for the next 20–30 min and ended with a 10-min cooling period of stretching exercises. The exercises were performed individually.

#### 2.3.1. Comprehensive Corrective Exercise Program

**Figure 2:**
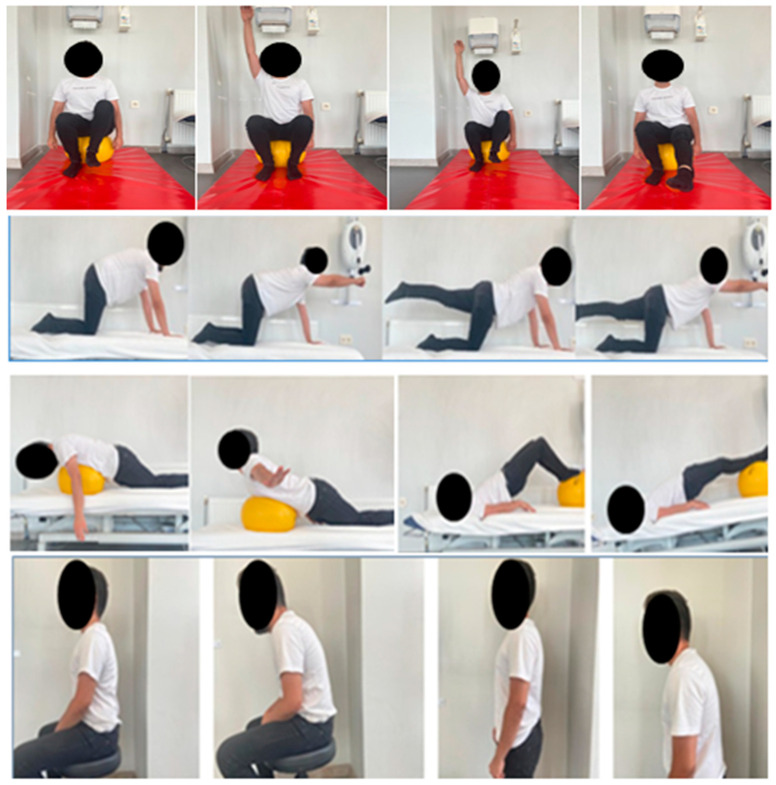
Examples of comprehensive corrective exercise and postural perception training program.

**Corrective exercises:** Chin tuck, stretching of neck extensor muscles and pectoral muscle groups in standing and supine positions, and postural perception training were applied for 12 weeks (see Appendix B). In the first and second weeks, stretching exercises were applied for 30 sec with 3 repetitions, followed by exercises in 2 sets with 5 repetitions. The number of repetitions was increased to 10 at weeks 3 and 4. Additionally, from week 5 to week 8, supine bridging on an unstable surface with knees flexed, unilateral lifting of the arms and feet in a crawling position, activation of the transversus abdominus muscle and unilateral arm and leg movements in a sitting position on an unstable surface, and the cat-camel exercise were performed in 3 sets with 10 repetitions. From week 9 to week 12, supine bridging on an unstable surface with knees extended, activation of the transversus abdominus muscle and contralateral arm and leg movements in a sitting position on an unstable surface, and ipsilateral–contralateral raising of arms and legs in a crawling position were performed in 3 sets with 15 repetitions (Figure 2).

**Postural perception training:** For PPT, the subjects were informed and trained about correct posture at the beginning of the program. As part of PPT, the subjects were asked to recognize their habitual postures and to adjust their sitting and standing positions to achieve a good posture (alignment of the head with the pelvis, pulling the shoulder blades together toward the spine (scapular retraction), chin slightly tucked in, thoracic extension, and relative increase in lordosis. During the 12-week training, the subjects were asked to assume a poor posture and then a good posture (3–5 times each) in sitting and standing positions in front of a mirror at each session. The subjects were questioned about how many times per week they noticed and corrected their poor posture in their daily lives, and the data were recorded (Figure 2).

#### 2.3.2. Thoracic Exercise Program

**Figure 3:**
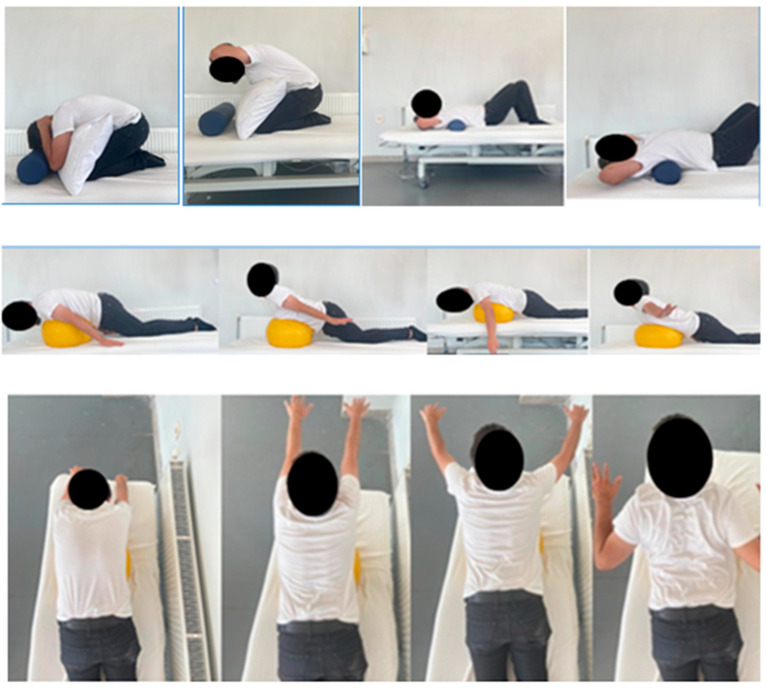
Examples of thoracic exercises.

Stretching of pectoral muscle groups and thoracic self-mobilization in standing and supine positions were applied for 12 weeks. During weeks 1–4, the set–rep numbers of stretching and exercises were the same as those of the CCEP program (Figure 3). Self-mobilization was performed for 1 min with 2 reps. T, Y, W and I exercises in a prone position and cat-camel exercises were performed in 3 sets with 10 reps from week 5 to week 8 and in 3 sets with 15 reps from week 9 to week 12 (see Appendix B).

### 2.4. Statistical Analysis

The study data were analyzed using SPSS (Statistical Package for Social Sciences; IBM Corp., Armonk, NY, USA) software, version 21.0. The Shapiro–Wilk test was used to check the normality of the data distribution. Repeated-measures ANOVA and Bonferroni post hoc tests were used to evaluate the difference between the measurements at different timepoints (group, time, time × group). Pre-test/post-test comparisons were performed using the paired samples *t*-test. The statistical significance level was set at *p* < 0.05. Eta squared was used to estimate the effect size. The effect size was interpreted as follows: small if 0.01, medium if 0.06, large if 0.14 [55]. The sample size was calculated using G × Power. 

## 3. Results

Before training, age, height, body weight and body mass index were similar among the groups (*p* > 0.05) (Table 1).

After training, a significant difference was observed among the groups in the kyphosis angle (*p* < 0.01). PPT values showed a significant difference in the CCEP group but not in other groups (*p* < 0.01). The Romberg index value did not differ among the groups (*p* > 0.05) (Table 2). A reduction in the kyphosis angle was observed in the CCEP (8.93°) and TEP (4.33°) groups upon within-group analyses (*p* < 0.01), whereas the kyphosis angle remained unchanged in the CG (*p* > 0.05). Post-intervention PPT and RI values showed changes in the CCEP group (*p* < 0.01) but did not change in the control and TEP groups (*p* > 0.05). The Romberg index (balance) improved in the CCEP group as shown by within- group analysis (*p* < 0.01) (Table 2). The effect size was large (0.254) for the kyphosis angle, large (0.385) for PPT, and small (0.041) for the RI. 

## 4. Discussion

The aim of this study was to investigate the effects of a comprehensive corrective exercise program on the kyphosis angle and balance in male adolescents with thoracic kyphosis. The results of our study showed a reduction in the thoracic kyphosis (TK) angle in the CCEP and TEP groups but not in the control group (CG). A greater reduction in the TK angle was found in the CCEP group than in the TEP group. The CCEP was more effective in improving balance versus the TEP. As a result, the CCEP, which included corrective exercises plus PPT, provided improvement in TK and balance.

Corrective exercise programs for TK are used to protect the spine during static/dynamic loading and to achieve ideal spinal alignment [38]. Feng et al. investigated the effects of a corrective functional exercise program on postural thoracic kyphosis in 164 adolescents with a TK angle of >40° versus a general exercise program designed in accordance with the curriculum. The corrective functional exercise program consisted of neck extension, pectoral stretching, bridging and pelvic tilt, thoracic rotation and extension, and cat–camel exercises. Before starting exercises, the subjects in the functional exercise group were instructed to stand in the same vertical plane as the tragus and lateral malleolus as part of the postural training. The subjects were asked to maintain this correct posture during the exercises, along with breathing exercises. The general exercise program consisted of sit-ups, push-ups, a 50 m run and squat exercises. Improvement in the kyphosis angle was reported in both groups, with greater improvement noted in those receiving the corrective functional exercise program [6]. Seidi et al. reported that comprehensive corrective exercises were significantly more effective than local corrective exercises in improving the kyphosis angle in young adults [30]. The favorable effects of a 12-week exercise program including spinal mobility, strengthening and alignment on kyphosis have been previously demonstrated [12]. In line with former studies, corrective exercises were applied in the current study. In addition, the spine was exposed to static/dynamic loads with Superman and ball exercises, which were synchronized with extremity movements. We think that, in this way, proper spine spinal alignment was achieved to withstand external loads that will occur during the activities of daily living.

Thoracic exercises have been reported to reduce kyphosis and increase spinal extensor muscle strength [24,27,56]. Thoracic extension exercises are used for facilitation and contractile tissue flexibility [27]. In male adults, stretching and muscle strengthening exercises for the thoracic region were shown to provide improvements in pain and thoracic kyphosis [56]. Improvement in the kyphosis angle was reported in a study examining the effects of a manual therapy and an exercise program [24]. Thoracic mobilization and extension exercises were shown to improve thoracic alignment [27]. In the present study, thoracic region-specific exercises (TEP) were applied to one of the study groups. A reduced TK angle was observed in the group receiving thoracic extension exercises. We believe that this reduction occurred due to increased muscle facilitation and strength with the use of thoracic extension exercises.

It has been reported that intervention programs aiming to correct posture in individuals with kyphosis should focus on stretching exercises for pectoral and hamstring muscles and strengthening exercises for core extensor muscles [6,24,57]. Stretching stimulates mechanoreceptors in the joints and muscles, and this stimulation can improve proprioception as well as muscle coordination and function [24,58,59]. Stretching exercises for neck, back and shoulder regions were reported to slow the progression of thoracic kyphosis [24]. In one study, a muscle strengthening program applied together with a muscle stretching program resulted in reductions in pain and thoracic kyphosis. A 13-week home-based exercise program, including pectoral stretching, strengthening of erector spinae muscles, cervical retraction and interscapular muscle strengthening, was shown to reduce TK angle by 3° [57]. In our study, a reduction in TK angle was observed in both groups receiving exercise programs. We think that the TK improvement was driven by improvements in muscle coordination and function via stimulation of the mechanoreceptors by stretching exercises applied as part of the exercise programs.

It has been suggested that mobility, posture and mobilization exercises should also be performed in order to maintain the beneficial effects of strengthening exercises over a long term. Mobilization and posture exercises provide co-activation of agonist and antagonist muscles [60]. Posture exercises combined with strengthening exercises have been reported to be effective in thoracic kyphosis [19]. Jang et al. reported improvement in the TK angle in the group receiving exercises to enhance perception of thoracic alignment mobility and stability plus breathing exercises compared to the group receiving a home-based exercise program [38]. Thoracic mobilization exercises were shown to have an effect on thoracic alignment [27]. Tarasi et al. reported that an exercise program involving spinal mobilization and alignment led to an improvement in the kyphosis angle in individuals with a TK angle of >42° [12]. In our study, improvement in TK was observed in the groups receiving spinal mobility exercise and also in the TEP group performing thoracic self-mobilization with foam rollers. We believe that the self-mobilization and mobility exercises used in our study affected thoracic kyphosis favorably by improving muscle coordination. 

Poor postural awareness is associated with habitual posture. It has been observed that poor habitual posture assumed during activities of daily living reduces the ability to sense, maintain and reposition the neutral spinal position. For this reason, we believe that it is more important for an individual to be aware of their posture in the first place in order to correct their bad posture. In the current study, PPT was employed to improve the awareness of posture in order to correct improper posture. Postural awareness improved in the CCEP group receiving PPT. Tarasi et al. applied postural training and spinal mobility, strengthening and alignment exercises to individuals with thoracic hyperkyphosis. They used visual materials that illustrate ideal spinal alignment while sitting, standing and sleeping. The authors reported beneficial effects of corrective exercises in combination with postural training on functional thoracic hyperkyphosis in young adults [12]. Katzman et al. investigated the effects of a TK-specific exercise program and postural training in individuals with a TK angle of ≥40°. For postural training, they used a manual of visuals depicting neutral spinal alignment. They concluded that a spine strengthening exercise together with a postural training program may be considered for older men and women with kyphosis [19]. Based on the data from an online survey of physiotherapists, Korakakis et al. reported that postural training was rated as “important” by 93.5% of the responders [61]. Studies have underscored the importance of restoring proprioception to correct posture and maintain proper posture. There is evidence that dynamic movements are more effective than static movements in improving proprioception [62]. In the current study, we did not evaluate proprioception. However, there are studies reporting that the Romberg Index (RI) value, which we used to assess balance, can be used as a measure of the proprioceptive system [53,63]. In light of the aforementioned data, it can be suggested that in the present study, proprioception improved through dynamic application of the PPT in sitting and standing positions. It can also be argued that postural awareness improved as a result of the improvement in proprioception. We think that improved postural awareness enables an individual to assume a correct posture more often in daily life. As a matter of fact, the findings of our study support this argument. The group receiving PPT (CCEP) showed an increase in the number of times/week the posture was noticed and corrected. 

In the present study, greater improvement of balance was observed in the CCEP group than in other groups, but the difference was not significant. Deviation of one of the spinal segments may shift the center of gravity away from the line of gravity. Increased thoracic kyphosis causes the gravity line to shift more anteriorly than in a normal stance. Displacement of the gravity line places greater strain on the vertebrae anteriorly and exceeds the limits of stability required for functional activities. As a result, the balanced movements of the trunk may be impaired, with greater muscle activity needed to stabilize the spine [63]. Beneficial effects of a 12-week kyphosis corrective exercise program on balance were shown in individuals with kyphosis angles of ≥50° [31]. An 8-week tailored exercise program (thoracic rotation and extension, scapular retraction, and external rotation and arm elevation) was reported to improve balance in individuals with TK angles of >40° [38]. In kyphotic individuals, exercises that increase the flexibility of the pelvic and shoulder girdle and strengthen core extensor muscles were reported to be effective in improving balance [64]. It was reported that a 4–week aerobic exercise program improved static and dynamic balance parameters in patients with osteoporosis [65]. In general, balance impairment has been associated with an increased risk of falls. Accordingly, the majority of studies have been conducted in the older population [33,34]. In contrast, our study involved a male adolescent population with thoracic kyphosis. We think that, in the current study, the exercises applied in the CCEP group increased muscle strength and provided correct alignment of the spine against external loads. In addition, we believe that PPT contributed to the perception of the neutral position of the spine, hence the gravity line, by improving postural awareness. It can be suggested that balance improvement in the CCEP group occurred as a result of these factors.

This is the first study to investigate the effects of a CCEP on thoracic kyphosis and balance in male adolescents. This study also compared a CCEP with a TEP. Improvement of TK was observed with both exercise programs, but the CCEP group showed greater improvement. Postural awareness improved only in the CCEP group. The differences in the thoracic kyphosis angle and postural awareness were associated with a large effect size. While balance did not differ among the groups after intervention, the CCEP group showed an improvement in balance upon within-group analysis. The difference in balance measurements was associated with a small effect size. As such, it can be said that a CCEP is effective in improving the TK angle and postural awareness. However, it cannot be suggested that a CCEP has a substantially different impact on balance compared to other methods.

### Limitations of the Study

PPT training improved posture by enhancing proprioception in this study. However, the lack of proprioceptive measurements should be noted as a limitation of our study. Muscle strength and endurance tests were not performed in the study. Assessment of strength and endurance of the trunk extensor muscles would have provided data in support of the reduction in the angle of kyphosis. In addition, gender–specific effects of the exercise programs applied in our study were not examined since only boys were included. The long-term effects of the exercise programs and PPT used in this study were not evaluated.

## 5. Conclusions

A comprehensive corrective exercise program provided improvement in kyphosis and postural awareness with a large effect size. This study aimed to improve poor posture and postural awareness with postural perception training, and achievement can be observed after 12 weeks of training in the CCEP group. However, further studies are required to demonstrate the effectiveness of a CCEP in diverse populations and the long-term effect of PPT. Corrective exercise with the inclusion of PPT can improve individuals’ posture and TK angle to correct thoracic kyphosis.

## Figures and Tables

**Table 1:** Demographic characteristics of the groups.

Demographic Characteristics	CCEP (*n* = 21) X ± SD (Min–Max)	TEP (*n* = 22) X ± SD (Min–Max)	CG (*n* = 19) X ± SD (Min–Max)	*p*
Age (years)	14.7 ± 1.3 (13–18)	13.5 ± 1.1 (12–16)	13.9 ± 1.9 (10–17)	0.650
Height (cm)	170.5 ± 8.6 (147–185	169 ± 5.8 (157–178)	168.3 ± 10.6 (145–187)	0.528
Body weight (kg)	61.8 ± 11.8 (39–82)	62.9 ± 11.8 (38–80)	61.9 ± 12.6 (40–88)	0.893
BMI (kg/m^2^)	21.5 ± 3.2 (14.1–25.8)	21.7 ± 3.5 (14.1–27)	21.7 ± 2.8 (13.8–25.6)	0.748

*p* < 0.05, *p*: Among groups, one-way analysis of variance (ANOVA). BMI: body mass index; CCEP: comprehensive corrective exercise program; TEP: thoracic exercise program; CG: control group; Min: minimum; Max: maximum; SD: standard deviation; X: mean.

**Table 2:** Comparisons of post-intervention thoracic kyphosis angle, postural perception training and balance measurements within and among the groups.

	CCEP				TEP				CG						
	Pre X ± SD	Post X ± SD	t	p	Pre X ± SD	Post X ± SD	t	p	Pre X ± SD	Post X ± SD	t	p	p × t (eta^2^)	p G × t (eta^2^)	p G (eta^2^)
Flexible Ruler (KA)	34.10 ± 6.03	25.17 ± 3.83	5.128	0.000 **	32.98 ± 6.38	28.65 ± 5.90	4.064	0.001 **	34.94 ± 3.87	35.28 ± 4.46	0.217	0.830	0.000 * (0.304)	0.000 * (0.247)	0.000 * (0.254)
PPT	2.14 ± 2.54	12.62 ± 4.07	11.555	0.000 **	3.18 ± 3.63	3.41 ± 3.23	0.439	0.665	2.63 ± 2.57	2.11 ± 2.54	1.455	0.163	0.000 * (0.580)	0.000 * (0.753	0.000 * (0.385)
RI	146.30 ± 86.1	27.71 ± 25.39	4.122	0.006 **	68,11 ± 62,1	87,13 ± 175,23	0.299	0.775	55.75 ± 40.4	54.03 ± 45.63	0.065	0.952	0.209 (0.091)	0.085 (0.252)	0.699 (0.041)

p × t: time; p G × t: group*time; p G: group * *p* < 0.05 repeated-measures ANOVA, ** *p* < 0.05 paired samples *t*-test; CCEP: comprehensive corrective exercise program; TEP: thoracic exercise program; CG: control group; PPT: postural perception training; RI: Romberg index; KA: kyphosis angle.

## Data Availability

The data that support the findings of this study are available from the corresponding author, [Gönül Elpeze], upon reasonable request.

## Appendix A. Exercise Schedule

**Weeks**	**Exercises**	**Exercise intensity**
Weeks 1–2	Stretching exercises	30 s/3 reps
	Self-mobilization	1 min/2 reps
	Exercises	5 × 2
Weeks 3–4	Stretching exercises	30 s/3 reps
	Self-mobilization	1 min/2 reps
	Exercises	10 × 2
Weeks 5–8	Stretching exercises	30 s/3 reps
	Self-mobilization	1 min/2 reps
	Exercises	10 × 3
Weeks 9–12	Stretching exercises	30 s/3 reps
	Self-mobilization	1 min/2 reps
	Exercises	15 × 3

## Appendix B. Comprehensive Corrective Exercise Program and Postural Perception Training Program

**Weeks**	**Comprehensive Corrective Exercise Program Plus Postural Perception Training Program (CCEP)**
Weeks 1–4	Chin-tuck exercises
	Stretching neck extensor muscles
	Stretching pectoral muscle groups in standing and supine positions
	Supine bridging on a stable surface
	Postural perception training
Weeks 5–8	Chin-tuck exercises
	Supine bridging with knees flexed on an unstable surface (ball)
	Unilateral lifting of the arms and legs in crawling position
	Activation of the transversus abdominus muscle and unilateral arm and leg movements in sitting position on an unstable surface
	Cat–camel exercise
	Stretching pectoral muscle groups in standing and supine positions
	Postural perception training
Weeks 9–12	Chin-tuck exercise
	Supine bridging with knees extended on an unstable surface
	Ipsilateral/contralateral raising of arms and legs in crawling position
	Activation of the transversus abdominus muscle and contralateral arm and leg movements in sitting position on an unstable surface
	Cat–camel exercise
	Stretching pectoral muscle groups in standing and supine positions
	Postural perception training
	**Thoracic Exercise Program (TEP)**
Weeks 1–4	Thoracic extension exercise in kneeling position
	Stretching pectoral muscle groups in standing and supine positions
	Self-mobilization
Weeks 5–8	T exercise in prone position
	Y exercise in prone position
	W exercise in prone position
	I exercise in prone position
	Cat–camel exercise
	Stretching pectoral muscle groups in standing and supine positions
	Self-mobilization
Weeks 9–12	T exercise in prone position
	Y exercise in prone position
	W exercise in prone position
	I exercise in prone position
	Cat–camel exercise
	Stretching pectoral muscle groups in standing and supine positions
	Self-mobilization
